# Alteration in Mir-21/PTEN Expression Modulates Gefitinib Resistance in Non-Small Cell Lung Cancer

**DOI:** 10.1371/journal.pone.0103305

**Published:** 2014-07-24

**Authors:** Hua Shen, Fang Zhu, Jinyuan Liu, Tongpeng Xu, Dong Pei, Rong Wang, Yingying Qian, Qi Li, Lin Wang, Zhumei Shi, Jitai Zheng, Qiudan Chen, Binghua Jiang, Yongqian Shu

**Affiliations:** 1 Department of Oncology, First Affiliated Hospital of Nanjing Medical University, Nanjing, Jiangsu Province, China; 2 Department of Cardiothoracic Surgery, First Affiliated Hospital of Nanjing Medical University, Nanjing, Jiangsu Province, China; 3 Department of Pathology, Cancer Center, Nanjing Medical University, Nanjing, Jiangsu Province, China; Seoul National University, Republic of Korea

## Abstract

Resistance to TKI treatment is a major obstacle in effective treatment of NSCLC. Besides EGFR mutation status, the mechanisms involved are largely unknown. Some evidence supports a role for microRNA 21 in modulating drug sensitivity of chemotherapy but its role in NSCLC TKI resistance still remains unexplored. This study aimed to investigate whether NSCLC miR-21 mediated resistance to TKIs also results from Pten targeting. Here, we show miR-21 promotes cancer by negatively regulating Pten expression in human NSCLC tissues: high miR-21 expression levels were associated with shorter DFS in 47 NSCLC patients; high miR-21/low Pten expression levels indicated a poor TKI clinical response and shorter overall survival in another 46 NSCLC patients undergoing TKI treatment. *In vitro* assays showed that miR-21 was up-regulated concomitantly to down-regulation of Pten in pc-9/GR cells in comparison with pc-9 cells. Moreover, over-expression of miR-21 significantly decreased gefitinib sensitivity by down-regulating Pten expression and activating Akt and ERK pathways in pc-9 cells, while miR-21 knockdown dramatically restored gefitinib sensitivity of pc-9/GR cells by up-regulation of Pten expression and inactivation of AKT and ERK pathways, *in vivo* and *in vitro*. We propose alteration of miR-21/Pten expression as a novel mechanism for TKI resistance in NSCLC cancer. Our findings provide a new basis for using miR 21/Pten-based therapeutic strategies to reverse gefitinib resistance in NSCLC.

## Introduction

Non-small cell lung cancer (NSCLC) is the leading cause of cancer related death worldwide. [1] Unfortunately, in spite of advances in early detection of cancer, the majority of patients with NSCLC are diagnosed with advanced-stage disease, resulting in poor prognosis with a median survival of only 10–12 months. [2], [3] The development of drugs that target the epidermal growth factor receptor (EGFR), such as EGFR-TKIs (gefitinib and erlotinib) has improved the efficacy of NSCLC therapy. Indeed, the presence of activating EGFR exon mutations plays a key role in predicting the therapeutic efficacy of EGFR-TKIs. [2] These activating EGFR mutations often significantly correlate with an adenocarcinoma-associated histology, smoking status, gender, and ethnicity. [3] Thus, EGFR-TKIs have been recommended as the first line therapy for NSCLC patients with EGFR mutations. Unfortunately, their clinical efficacy is limited by the development of acquired resistance: most patients who initially respond would subsequently experience disease progression with continuous use of TKI for about 9–11 months; [4] about 50% of patients who initially respond to EGFR-TKI acquire resistance to EGFR-TKI, while EGFR T790M mutation in exon20 accounts for 50% of this acquired resistance; [4] another 20% of TKI acquired resistance results from MET amplification. [2] It is still unclear how the remaining 30% of patients develop acquired resistance.

MicroRNA (miRNA), known to intrinsically suppress mRNA by pairing with the 3′-untranslated region (UTR) of the mRNA was shown to negatively modulate expression of targeted genes. [5], [6] As genes regulators, miRNA regulate about one third of genes and play important roles in cellular functions including proliferation, growth, differentiation and apoptosis. [7], [8] In recent years, the crucial role of miRNAs in carcinogenesis have been demonstrated. [9] Moreover, some miRNAs function as tumor suppressors *in vitro.* [10], [11] Therefore, over-expression of such miRNAs may suppress the target proteins which function as carcinogenic factors. [7], [12] In contrast to short interfering RNA, miRNAs is believed to regulate the same pathway at multiple levels. [10] MiR-21 is an important oncogenic miRNA, closely related to tumor growth and metastasis. [13], [14] Indeed, expression of miR-21 was shown to be associated with poor prognosis and chemo-sensitivity in colon adenocarcinoma. [15], [16] In addition, anti-miR-21 suppressed cell growth of breast cancer through down-regulation of the antiapoptotic factor, B-cell lymphoma 2 (Bcl-2). [17], [18] These data, taken together, support an important role of altered miR-21 expression during tumor development. So we investigated whether miR-21 modulates TKI sensitivity in NSCLC patients as well.

We found that up-regulation of miR-21 and down-regulation of Pten in 47 NSCLC tumor tissues compared with their adjacent normal tissues, and that their expression levels negatively correlated. miR-21 expression correlated with shorter DFS in the 47 NSCLC patients. In addition, our data showed a good correlation between high miR-21/low Pten expression levels and poor TKI sensitivity with short overall survival in 46 NSCLC patients undergoing TKI treatment. Therefore, we hypothesized that alteration of miR-21-Pten expression modulates TKI sensitivity in lung cancer cells. We found that miR-21 was up-regulated concomittantly with down-regulation of Pten in pc-9/GR cells compared with pc-9 cells, *in vitro*. Furthermore, over-expression of miR-21 significantly decreased gefitinib sensitivity through down-regulation of Pten expression and activation of Akt and ERK pathways, while knockdown of miR-21 dramatically restored gefitinib sensitivity of pc-9/GR cells by up-regulating Pten expression and inactivating AKT and ERK signaling pathways, both *in vivo* and *in vitro*.

## Materials and Methods

### Materials

Gefitinib was a kind gift of AstraZeneca (Tocris, United Kingdom). Antibodies against PTEN, phospho-ERK1/2, phospho-AKT (Ser-473), and total AKT were purchased from Cell Signaling Technology (Beverly, MA). Antibodies against ERK2 were purchased from Santa Cruz Biotechnology (CA, USA). Antibodies against GAPDH were obtained from Kangcheng Company (Shanghai, China). Lipofectamine and TRIzol reagents were purchased from Life Technologies (Grand Island, NY, USA). MiR-21 mimics and miR-21 inhibitor were provided by GenePharma (shanghai, China). The High Capacity RNA-to cDNA Kit and Power SYBR Green PCR Master Mix were purchased from Applied Biosystems (Carlsbad, CA). The PC-9 cell lines were purchased from the Type Culture Collection of the Chinese Academy of Sciences, Shanghai, China. The PC-9 gefitinib resistant cell line (PC-9/GR), which was induced by exposition of PC-9 cells to increasing concentrations of gefitinib as reported previously [19], [20], was kindly provided by Department of Oncology, Shanghai Pulmonary Hospital, Tongji University, Shanghai.

### Clinical specimens

Paired human NSCLC specimens (n = 47) and matched normal adjacent tissue samples were collected from patients undergoing standard surgical procedures in the First Affiliated Hospital of Nanjing Medical University, Jiangsu (China), with written informed consent of patients. A part of each tissue sample was immediately snap-frozen in liquid nitrogen while the other part was fixed in formalin for histological examination.

Paraffin embedded samples of patients with NSCLC (n = 46), who were receiving EGFR-TKI treatment, were collected from pathology department of First Affiliated Hospital of Nanjing Medical University, Jiangsu (China), with written informed consent of the patients. All samples were histologically classified and graded according to TNM stage by a blinded clinical pathologist. All experimental protocols were approved by the Institutional Review Committee of the first affiliated hospital of Nanjing Medical University, Nanjing (China).

### Cell culture

Human pc-9 and pc-9/GR cell lines, and HEK293T cells were cultured in RPMI and DMEM, respectively, supplemented with 10% fetal bovine serum, 100 units/ml penicillin, and 100 µg/ml streptomycin at 37°C in a humidified environment containing 5% CO_2_.

### Cell transfection

Hsa-miR-21 or hsa-miR-NC mimics were transfected into PC-9 cells using the lipofectamine reagent (Life Technologies), according to the manufacturer’s instructions. The final concentrations of hsa-miR-21 or hsa-miR-NC mimics for the transfection were 40 nM. A similar procedure was used to transfect miR-21 inhibitor (40 nM) or NC into PC-9/GR cells.

### Cell viability assay

Pc-9 and Pc-9/GR cells were seeded into 96-well plates at a density of 4×10^3^ cells/well and allowed to adhere overnight. In parallel, Pc-9 cells were seeded into 96-well plates and transfected with miR-21 mimic and NC for 24 h, while pc-9/GR cells were transfected with miR-21 inhibitor. After cellular adhesion, gefitinib was added at a final concentration of 2.5–40 µmol/L. After 72 h incubation, cell viability was assessed using the Cell Counting Kit-8 (CCK-8) (Dojindo Laboratories), according to the manufacturer’s instructions. Each sample was plated in triplicate and three independent experiments were performed.

### Lentiviral packaging and stable cell line establishment

To stably knockdown miR-21 expression in pc-9/GR cells, the lentiviral packaging kit (Thermo Fisher Scientific) was used. Lentivirus carrying miR-21 inhibitor or negative control (miR-NC) was packaged following the manufacturer’s instructions. Green fluorescent protein (GFP) gene was inserted into the packaging system and co-expressed with miRNAs. Lentivirus was packaged in HEK293T cells and secreted into the medium. Pc-9/GR cells were infected by lentivirus carrying miR-21 inhibitor or miR-NC in the presence of polybrene (Sigma-Aldrich), and selected by puromycin (Sigma-Aldrich) for 2 weeks to obtain pc-9 GR/miR-21 inhibitor and pc-9 GR/miR-NC stable cell lines.

### Apoptosis assay

Apoptosis was measured as described previously [21]. Briefly, cells were trypsinized and labeled with Alexa Fluor 647 Annexin V (Biolegend) and 7-AAD (BD Pharmingen), and analyzed by flow cytometry. Cells were considered apoptotic when they were annexin V-positive and 7-AAD–negative.

### RNA isolation and quantitative real-time PCR (qRT-PCR) analysis

Total RNAs were extracted from cultured cells or human tissue specimens using TRIzol reagent according to the manufacturer’s instructions. To measure miR-21 expression levels, RNAs were transcribed by stem-loop RT primer using PrimeScript RT Reagent Kit (Takara) as previously described [21], [22]. The sequences of the RT primers were as follows: miR-21-RT, 5′-CTCAACTGGTGTCGTGGAGTCGGCAATTCAGTTGAG TCAACATC -3′; U6-RT, 5′-TGGTGTCGTGGAGTCG-3′. qRT-PCR was performed using SYBR Premix DimerEraser (Takara) on a 7900HT system (Applied Biosystems). miR-21 qPCR primers were: sense, 5′-ACACTCCAGCTGGG TAGCTTATCAGACTGA -3′; anti-sense, 5′- TGGTGTCGTGGAGTCG -3′. U6 snRNA qPCR primers were: sense, 5′-CTCGCTTCGGCAGCACA-3′; anti-sense, 5′-AACGCTTCACGAATTTGCGT-3′. The expression of miR-21 was normalized to the levels of U6 and the comparative cycle threshold method (2^−ΔΔCT^) was used with U6 as control.

### Protein extraction and western blotting

Cells or tissues were harvested and lysed on ice for 30 minutes in RIPA buffer (Beyotime) supplemented with 1 mM phenylmethylsulfonyl fluoride (PMSF). Lysates were subjected to Western blotting assay as described previously [23] and detected with antibodies against PTEN, phospho-AKT (Ser-473), total AKT, phospho-ERK, total ERK, GAPDH.

### Immunohistochemistry (IHC)

Formalin-fixed, paraffin-embedded tissues collected from patients with NSCLC (n = 46), were sectioned at 5 µm, and incubated with antibodies against Pten (Cell Signaling Technology). The assessment of IHC signals was performed by two experienced pathologists in a blinded manner. Five high-power fields (200×) were randomly selected in every sample. Percentages of tumor cells were categorized into five semi-quantitative classes: 0 (≤5% positive cells), 1 (6%–25% positive cells), 2 (26%–50% positive cells), 3 (51%–75% positive cells), and 4 (>76% positive cells). Intensity of staining was also semi-quantitatively determined on a scale of 0–3 as follows: 0 (negative), 1 (weakly positive), 2 (moderately positive), and 3 (strongly positive). The products of percentage scores by intensity of staining yielded final IHC scores: 0 (negative),+(1–4),++(5–8), and+++(9–12) as previously described [24].

### In situ hybridization

ISH was conducted on paraffin embedding samples of NSCLC to investigate the clinical response to TKI and miR-21 expression. Briefly, slides were treated and hybridized with 10 pmol probe (LNA-modified and DIG labeled oligonucleotide; Exiqon) complementary to miR-21, according to the manufacturer’s instructions. After incubation with anti-DIG-HRP fab fragments conjugated to horseradish peroxidase, the hybridized probes were detected by incubation with 3′3-diaminobenzidine solution with nuclei counterstained with Carazzi’s haematoxylin. Staining patterns were analyzed by 3 experts and the proportion of positively stained tumor cells was graded as follows: 0 (no positive cells), 1 (<10% positive cells), 2 (10%–50% positive cells), 3 (>50%positive cells). Cells at each intensity of staining were recorded on a scale of 0 (no staining), 1(weak staining, light blue or yellow), 2 (moderate staining, blue or yellow), and 3 (strong staining, dark blue or yellow). For tumors that showed heterogeneous staining, the predominant pattern was considered for scoring. The staining index (SI) was calculated as proportion of positively stained tumor cells×staining intensity. Using this method, the expression of miR-21 was scored as 0, 1, 2, 3, 4, 6, or 9. In case of disagreement (score discrepancy>1), slides were reexamined and a consensus was reached by the experts.

### Xenograft tumor model in nude mice

For tumor growth assays, 6-week-old male nude mice (BALB/cA-nu (nu/nu) were purchased from SLAC Animal Center (Shanghai, China), and maintained in special pathogen-free (SPF) conditions. Protocols for animal experiments were approved by the Animal Welfare Committee of Nanjing Medical University. Aliquots of cells (4×10^6^) were suspended in 150 µl FBS-free RPMI DMEM medium and subcutaneously injected into posterior flank of nude mice (n = 6). Tumor size was measured using a Vernier caliper every seven days until they were sacrificed on day 35 after implantation and tumor volume was derived as 0.5×Length×Width^2^.

### Statistical analysis

Values were obtained from at least three independent experiments and presented as means ± SE. Student’s unpaired *t* test was performed and values were considered significantly different when *P*<0.05.

## Results

### Up-regulation of miR-21 and down-regulation of Pten negatively correlate with shorter DSF in tumor tissues from human NSCLC patients

**Figure 1:**
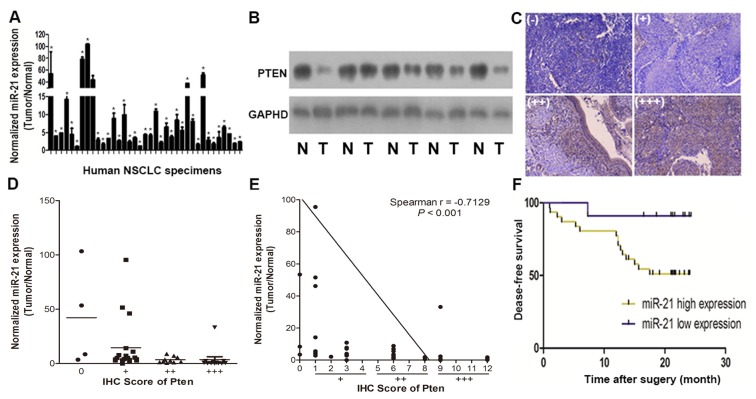
MiR-21/Pten expression levels in 47 pairs of NSCLC tissues and adjacent normal specimens, and relationship with disease free survival. (A) Expression levels of miR-21 were up-regulated in 37/47 pairs of NSCLC tissues relatively to adjacent normal specimens. MiR-21 were analyzed by stem-loop qRT-PCR, and normalized to the levels of U6. Fold changes were obtained by the ratio of miR-21 abundance in cancer tissues to that in adjacent normal tissues. *p<0.05 indicates significant difference comparing miR-21 expression in tumor tissues with adjacent normal tissues. (B) Representative Pten protein levels in NSCLC tissues (T) and adjacent normal specimens (N) assessed by immunoblot, GAPDH was used as loading control. (C) Pten Immunohistochemistry (IHC) results of NSCLC sections, 0 (negative), +(weakly positive),++(moderately positive), and+++(strongly positive). (D) Correlation analysis between Pten protein levels and miR-21 expression levels in NSCLC tissues. IHC scores were used to describe Pten protein levels in tumor tissues, evaluated by experienced pathologists in a blinded manner. (E) Linear regression curves of miR-21 expression and Pten protein levels. (F) Kaplan-Meier curves depicting Disease-free survival according to expression of miR-21. The cutoff value of the subgroups (high and low) of miR-21 expression level was the 50th percentile value.

To determine the expression levels of miR-21 and Pten protein in tumor tissues from human NSCLC patients, we assessed 47 pairs of cancer tumor specimens and adjacent normal tissues (Table S1) by qRT-PCR and found a significantly higher expression of miR-21 in 37/47 (78.7%) in tumor tissues in comparison with adjacent normal tissues (Fig. 1A). Since Pten is one of the important targets of miR-21, [25], [26] we investigated the relationship between expression of miR-21 and Pten in human NSCLC specimens both by immunoblot and immunohistochemistry (IHC). We found that Pten protein levels were significantly reduced in 34/47 (72.3%) tumor tissues compared with adjacent normal ones (Fig. 1B). In addition, the relative expression levels of Pten were evaluated by two experienced pathologists in a blinded manner, and marked with final IHC scores of 0 (negative),+(weak),++(moderate) and+++(strong) (Fig. 1C). Levels of miR-21 were reduced at high Pten intensity (Fig. 1D). In addition, scatter plot analysis showed an inverse correlation between miR-21 expression levels and IHC scores of Pten signals (Fig. 1E). These results demonstrated that miR-21 expression levels were inversely correlated with Pten levels in NSCLC tissues. To further evaluate the correlation between miR-21/pten expression levels and prognosis in NSCLC patients, we used Kaplan-Meier survival analysis and log-rank test to assess the normalized miR-21 expression levels (tumor/normal) and Disease free survival (DFS). The results showed that patients with high miR-21 expression levels had a shorter disease free survival (DFS) compared with patients with low miR-21 expression (Fig. 1F).

### Up-regulation of miR-21 and down-regulation of Pten protein are associated with poor TKI sensitivity and shorter overall survival in tumor tissues of human NSCLC patients undergoing TKI treatment

**Figure 2:**
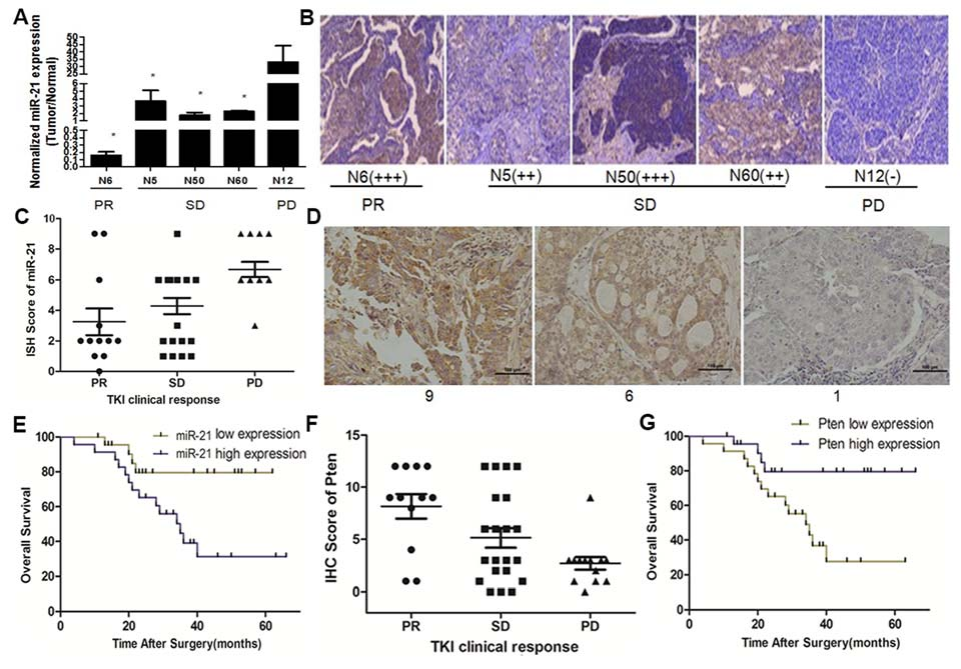
MiR-21/Pten expression levels and their relationship with TKI sensitivity and overall survival in NSCLC patients undergoing TKIs treatment. (A) Clinical response of TKI and miR-21 expression levels in 5 NSCLC patients evaluated by q-RT PCR. (B) Clinical response of TKI and Pten expression levels in 5 NSCLC patients tested by Immunohistochemistry. PR (partial response) and SD (stable disease) patients had relatively lower miR-21 expression levels and higher Pten expression levels compared with PD (progressive disease) patients. (*p<0.05). (C) Correlation analysis performed between miR-21 expression levels and clinical response to TKIs. (D) Representative miR-21 levels and scores in NSCLC tissues undergoing TKI treatment evaluated by in situ hybridization. (E) Kaplan-Meier curves depicting overall survival according to expression of miR-21 in 46 NSCLC patients undergoing TKIs treatment. (F) Correlation analysis performed between Pten expression levels and clinical response to TKIs. (E) Kaplan-Meier curves depicting overall survival according to expression of Pten in 46 NSCLC patients undergoing TKIs treatment.

Previous studies have demonstrated that miR-21 induces cisplatin resistance in NSCLC [27]. In addition, Wang et al. found that miR-214 regulates the acquired resistance to gefitinib via the PTEN/AKT Pathway in EGFR-mutant cell lines [28]. Given the potential effect of miRNA in modulating drug sensitivity, we investigated whether alteration of miR-21/Pten expression correlates with TKI sensitivity. After follow up of clinical treatment of the 47 NSCLC patients, we found only 5 patients that used TKI treatment. One patient showing a partial response(PR) showed miR-21 expression level (tumor/normal) of only 0.16; three patients with stable disease (SD) displayed average miR-21 expression levels of 2.57; one patient with a progressive disease (PD) showed high miR-21 expression at 33.15 (Fig. 2A). IHC assays on tissues obtained from these five patients showed that Pten expression levels negatively correlated with clinical response of TKI: one PD patient tissue showed Pten expression of (–); Pten expression in the remaining four patients with PR and SD ranged from (++) to (+++) (Fig. 2B). To further confirm our findings, we assessed miR-21 expression levels using ISH assays (Fig. 2D) and Pten expression levels by IHC on paraffin embedded samples from 46 NSCLC patients treated with TKI(gefitinib or erlotinib) as treatment and followed up their clinical response and survival status (Table S2). As expected, miR-21 expression levels in PD patients were significantly higher than those in PR and SD groups(Fig. 2C). Higher miR-21 expression levels also indicated shorter overall survival in TKI treated patients (Fig. 2E). In addition, Pten expression levels in PD patients was significantly lower than in PR and SD patient groups (Fig. 2F), and higher Pten expression correlated with longer overall survival in TKI treated patients (Fig. 2G). These findings are all in agreement and suggest involvement of high miR-21/low Pten expression in modulation of TKI sensitivity in NSCLC.

### PC9/GR cells show significant resistance to gefitinib, up-regulation of miR-21, down-regulation of Pten and activation of AKT, ERK compared with PC9 cells

**Figure 3:**
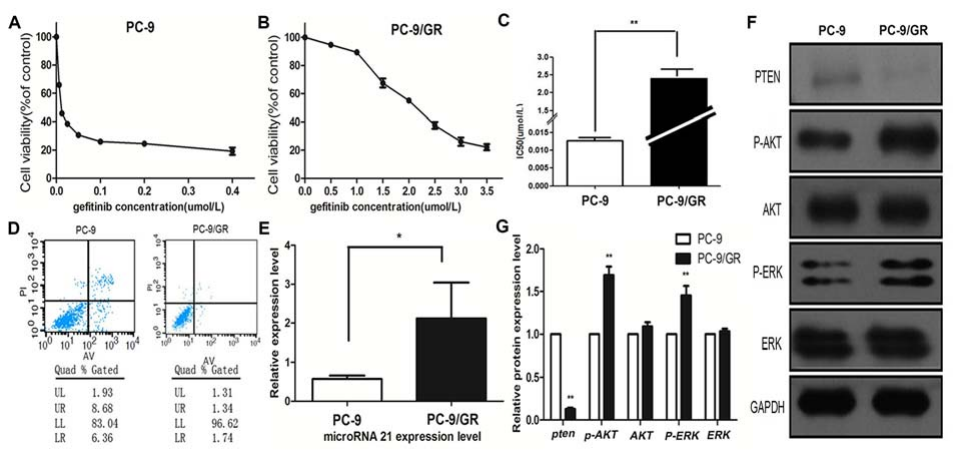
pc-9/GR cells show gefitinib resistance properties, up-regulation of miR-21 and down-regulation of PTEN compared with pc-9 cells. (A, B) pc-9 and pc-9/GR cells were treated with gefitinib for 72 h in 0.1% serum-containing RPMI. Cell growth was measured by CCK-8 assay in triplicate. Cell growth at 72 h is plotted against gefitinib concentration. (C) gefitinib IC 50 in pc-9 and pc-9/GR cells with triplicate assays performed. **indicates p<0.01. (D) Apoptosis rates of pc-9/GR and pc-9 cells induced by gefitinib were evaluated by flow cytometry. pc-9/GR and pc-9 cells were treated with 1 µmol/L gefitinib for 72 hours before apoptosis evaluation. (E) Relative expression levels of miR-21 in pc-9 and pc-9/GR cells assessed by stem-loop qRT-PCR, and normalized to U6 levels. Triplicate assays were performed for each RNA sample. Significant differences are indicated by *(P<0.05). (F) Pten, p-AKT, AKT, p-ERK, ERK proteins in pc-9 and pc-9/GR cells analyzed by Western blot. (G) Quantitation of western blot analysis showed that Pten protein expression was reduced in PC-9/GR cell compared with PC-9 cells **P<0.01; p-AKT and p-ERK proteins expression were up-regulated in PC-9 GR cells compared with PC-9 cells **P<0.01; there were no significant differences of total AKT and total ERK proteins between PC-9 and PC-9/GR cells.

In order to test whether the effect of high miR-21/low Pten expression on modulation of TKI sensitivity, we selected pc-9, a TKI sensitive cell line, and the gefitinib resistant cell line PC-9/GR (kindly provided by Department of Oncology, Shanghai Pulmonary Hospital, Tongji University, Shanghai) were induced by exposition of PC-9 cell to increasing concentrations of gefitinib as reported previously. [19], [20] We used the CCK-8 assay to detect gefitinib sensitivity in pc-9 and pc-9 GR cells and found that pc-9 GR cells were resistant to gefitinib compared with pc-9 cells(Fig. 3A, Fig. 3B). The IC50 for gefitinib in PC9/GR cells were about 2.54 µmol/L, 200times higher than in PC9 cells (0.0127 µmol/L, P<0.001) (Fig. 3C). After 72 h of 1 µmol/L gefitinib treatment, we used Flow cytometry to assess gefitinib induced apoptosis rates. Our data showed apoptosis rates significantly higher in PC9 cells compared with PC9/GR (15.04% vs. 3.08%) (Fig. 3D). qRT-PCR data showed that miR-21 was 3.70-fold upregulated in PC9/GR cells compared with PC9 cells (Fig. 3E). Using western-blot assays, we showed Pten loss and activation of AKT and ERK in PC-9 GR cells compared with PC-9 cells, but did not show total AKT and total ERK expression difference between the two cell lines (Fig. 3F, 3G).

### Elevated expression of miR-21 reduced gefitinib sensitivity, enhanced invasive ability and reduced gefitinib induced apoptosis in pc-9 cells by down-regulating Pten and activating AKT and ERK pathways

**Figure 4:**
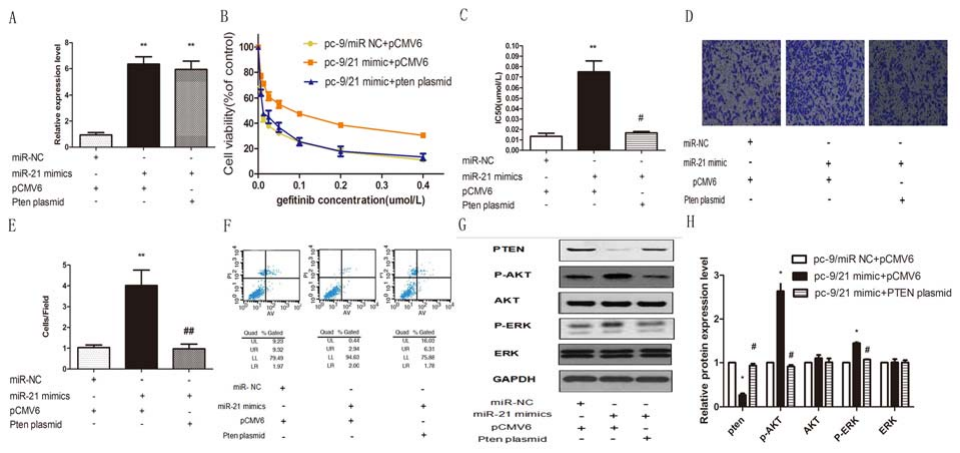
Effects of transient enhanced expression of miR-21 mimics and Pten rescue in Pc 9 cell lines. (A) Relative expression levels of miR-21 in pc-9/NC +pCMV 6 transfected cells, pc-9/miR-21 mimic+pCMV 6 transfected cells and pc-9/miR-21 mimic+Pten plasmid transfected cells were assessed by stem-loop qRT-PCR, and normalized to U6 levels. Triplicate assays were performed for each RNA sample. Significant differences are indicated by **(P<0.01). (B) gefitinib sensitivity of pc-9/NC +pCMV 6 transfected cells, pc-9/miR-21 mimic+pCMV 6 transfected cells and pc-9/miR-21 mimic+Pten plasmid transfected cells were evaluated by CCK-8 assay. (C) IC50 for gefitinib in pc-9/NC +pCMV 6 transfected cells, pc-9/miR-21 mimic+pCMV 6 transfected cells and pc-9/miR-21 mimic+Pten plasmid transfected cells. **P<0.001, #P<0.01. (D) pc-9/NC +pCMV 6 transfected cells, pc-9/miR-21 mimic+pCMV 6 transfected cells and pc-9/miR-21 mimic+Pten plasmid transfected cells were used for cell invasion assays. Invasion cells stained by 0.1% crystal violet were counted 24 h after plating. Upper panels: representative photographs of invasive cells (×200). (E) Number of invasive cells per field was obtained from at least three replicate wells and represented by mean ± SD (bottom graph). **indicates significant difference between pc-9/NC+pCMV 6 transfected cells and pc-9/miR-21 mimic +pCMV 6 transfected cells (P<0.01). ## indicates significant difference between pc-9/miR-21 mimic +pCMV 6 transfected cells and pc-9/miR-21 mimic+Pten plasmid transfected cells (P<0.01). (F) Apoptosis rates of pc-9/NC +pCMV 6 transfected cells, pc-9/miR-21 mimic+pCMV 6 transfected cells and pc-9/miR-21 mimic+Pten plasmid transfected cells induced by gefitinib were evaluated by flow cytometry. All cells were treated with 1 µmol/L gefitinib for 72 hours before apoptosis evaluation. (G) Pten, p-AKT, AKT, p-ERK, and ERK proteins of pc-9/NC +pCMV 6 transfected cells, pc-9/miR-21 mimic+pCMV 6 transfected cells and pc-9/miR-21 mimic+Pten plasmid transfected cells were analyzed by Western blot. (H) Quantification of western blot analysis showed down-regulation of Pten protein in PC-9/21 mimics +pCMV 6 transfected cells compared with PC-9/NC+pCMV 6 transfected cells (*P<0.05) and pc-9/miR-21 mimic+Pten plasmid transfected cells (#P<0.05); p-AKT and p-ERK proteins expression were up-regulated in PC-9/21 mimics +pCMV 6 transfected cells compared with pc-9/NC +pCMV 6 transfected cells (*P<0.05) and pc-9/miR-21 mimic+Pten plasmid transfected cells (#P<0.05). There were no significant differences in total AKT and total ERK proteins among the three cells.

Our results described above showed that miR-21 was up-regulated and Pten was down-regulated in PC-9 GR cells compared with PC-9 cells. Therefore, we hypothesized that miR-21/Pten expression alteration plays an important role in modulating gefitinib sensitivity in pc-9 cells and pc-9/GR cells. We transfected pc-9 cells with miR-21 mimics and then co-transfected these cells with Pten plasmid to observe whether Pten could rescue miR-21 induced biological changes in pc-9 cells. qPCR data confirmed that miR-21 expression was increased significantly in miR-21 mimic transfected cells compared with the NC transfected cells, **p<0.01 (Fig. 4A). Then we used the CCK-8 assay kit to detect gefitinib sensitivity 24 h after transfection. As expected, miR-21 did reduce gefitinib sensitivity in pc-9 cells and over-expression Pten could rescue this gefitinib sensitivity change. **p<0.01 (Fig. 4B, 4C). To observe other parameter changes after miR-21 transfection, we performed a transwell assay to determine the influence of miR-21 on invasive ability. We found that miR-21 transfected pc-9 cells were more invasive than the NC group and over-expression Pten could rescue this invasive ability **p<0.01 (Fig. 4D, 4E). In order to detect the impact of miR-21 on gefitinib induced apoptosis, the cells described above were treated with 1 µmol/L gefitinib for 72 hours. Using flow cytometry, we found that in pc-9 cells, a marked decrease in apoptosis was observed in miR-21 mimic transfected cells compared with NC transfected cells and miR-21 mimic and Pten plasmid co-transfected cells after gefitinib treatment(Fig. 4F). To better understand the molecular mechanisms involved in miR-21 induced gefitinib resistance in pc-9 cells, western-blot assays were used to assess the expression of related proteins. In pc-9 cells, elevated expression of miR-21 resulted in repression of Pten expression and activation of p-AKT and p-ERK in comparison with the NC group, but did not change total protein expression of AKT and EKR. Over-expression of Pten expression could rescue up-regulation of miR-21 induced activation of p-AKT and p-ERK. (Fig. 4G, 4H).

### Knockdown of miR-21 restored gefitinib sensitivity, reduced the invasive ability and enhanced gefitinib induced apoptosis in pc-9/GR cells by up-regulating Pten and inactivating the AKK and ERK pathways

**Figure 5:**
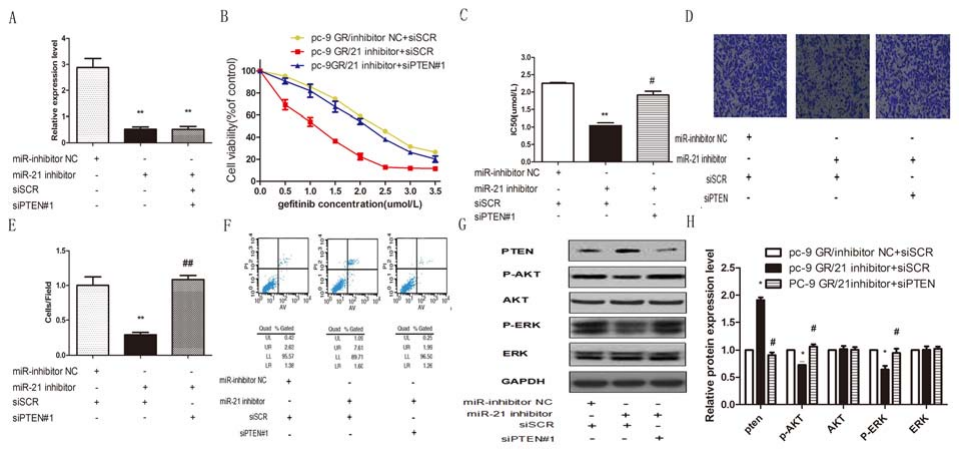
Effects of miR-21 knockdown and Pten down-regulation in Pc 9/GR cell lines. (A) Relative expression levels of miR-21 in pc-9 GR/inhibitor NC+scramble siRNA(siSCR) transfected cells, pc-9 GR/miR-21 inhibitor+scramble siRNA(siSCR) transfected cells and pc-9 GR/miR-21 inhibitor+Pten siRNA transfected cells were assessed by stem-loop qRT-PCR, and normalized to the levels of U6. Triplicate assays were performed for each RNA sample. Significant differences are indicated by **(P<0.01). (B) Gefitinib sensitivity of pc-9 GR/inhibitor NC+ scramble siRNA(siSCR) transfected cells, pc-9 GR/miR-21 inhibitor+scramble siRNA(siSCR) transfected cells and pc-9 GR/miR-21 inhibitor+Pten siRNA transfected cells were evaluated by CCK-8 assay. (C) IC50 values for gefitinib in PC9 GR/21 inhibitor+scramble siRNA(siSCR) transfected cells were significantly lower than in PC9 GR/inhibitor NC+ scramble siRNA(siSCR) transfected cells(**p<0.01) and pc-9 GR/miR-21 inhibitor+Pten siRNA transfected cells(#p<0.05). (D) pc-9 GR/inhibitor NC+ scramble siRNA(siSCR) transfected cells, pc-9 GR/miR-21 inhibitor+scramble siRNA(siSCR) transfected cells and pc-9 GR/miR-21 inhibitor+Pten siRNA transfected cells were used for cell invasion assays. Invasion cells stained by 0.1% crystal violet were counted 24 h after plating. Upper panels: representative photographs of invasive cells (×200). (E) Number of invasive cells per field was obtained from at least three replicate wells and represented by mean ± SD (bottom graph). **indicates significant difference between pc-9 GR/inhibitor NC+ scramble siRNA(siSCR) transfected cells and pc-9 GR/miR-21 inhibitor+scramble siRNA(siSCR) transfected cells (P<0.01). ## indicates significant difference between pc-9 GR/miR-21 inhibitor+scramble siRNA(siSCR) transfected cells and pc-9 GR/miR-21 inhibitor+Pten siRNA transfected cells (P<0.01). (F) Apoptosis rate of pc-9 GR/inhibitor NC+ scramble siRNA(siSCR) transfected cells, pc-9 GR/miR-21 inhibitor+scramble siRNA(siSCR) transfected cells and pc-9 GR/miR-21 inhibitor+Pten siRNA transfected cells induced by gefitinib were evaluated by flow cytometry. All cells were treated with 1 µmol/L gefitinib for 72 hours before apoptosis evaluation. (G) Pten, p-AKT, AKT, p-ERK, and ERK proteins in pc-9 GR/inhibitor NC+ scramble siRNA(siSCR) transfected cells, pc-9 GR/miR-21 inhibitor+scramble siRNA(siSCR) transfected cells and pc-9 GR/miR-21 inhibitor+Pten siRNA transfected cells were analyzed by Western blot. (H) Quantification of western blot showed up-regulated expression of Pten protein in PC-9 GR/21 inhibitor+scramble siRNA(siSCR) transfected cells compared with PC-9 GR/inhibitor NC+ scramble siRNA(siSCR) transfected cells (*P<0.05) and pc-9 GR/miR-21 inhibitor+Pten siRNA transfected cells(#P<0.05); p-AKT and p-ERK proteins expression was down-regulated in PC-9 GR/21 inhibitor+scramble siRNA(siSCR) transfected cells compared with PC-9 GR/inhibitor NC+ scramble siRNA(siSCR) transfected cells (*P<0.05) and pc-9 GR/miR-21 inhibitor+Pten siRNA transfected cells (#P<0.05). There were no significant differences in total AKT and total ERK proteins among the three cells.

To further confirm the function of miR-21 on gefitinib sensitivity regulation in pc-9/GR cells, pc-9/GR cells were transfected with miR-21 inhibitor to decrease miR-21 expression and then co-transfected these cells with Pten siRNA. We first tested the effect of Pten siRNA, we transfected pc-9/GR cells with two Pten siRNAs and scramble siRNA(siSCR). Both the WB and the qRT-PCR assays showed that siPTEN#1 could decrease PTEN protein (Fig. S1A), and mRNA(Fig. S1B) expression level more effectively than siPTEN#2. So we chose siPTEN#1 to do further experiment. qPCR results confirmed that miR-21 expression was decreased significantly in miR-21 inhibitor transfected cells compared with NC cells, **p<0.01 (Fig. 5A). CCK-8 assay data showed that miR-21 knockdown significantly decreased gefitinib sensitivity of pc-9/GR and reduced Pten expression level could restore gefitinib sensitivity of pc-9/GR **p<0.01 (Fig. 5B, 5C). Transwell assay data also showed that miR-21 knockdown reduced the invasive ability of pc-9/GR cells but it could be rescued by down-regulation of Pten **p<0.01 (Fig. 5D, 5E). Using flow cytometry, we showed that knockdown of miR-21 increased apoptosis rates in pc-9/GR cells and down-regulation of Pten could also restore this apoptosis rates change, when cells were treated with 1 µmol/L gefitinib for 72 hours (Fig. 5F).

Finally, western-blot data showed that knockdown of miRNA 21 in pc-9/GR cells increased Pten expression and inactivated p-AKT and p-ERK relatively to NC group, but did not affect total protein expression levels in AKT and EKR. Down-regulation of Pten expression could rescue knockdown miR-21 induced inactivation of p-AKT and p-ERK.(Fig. 5G, 5H).

### Anti-miR21 restored gefitinib sensitivity of pc-9/GR *in vivo*

**Figure 6:**
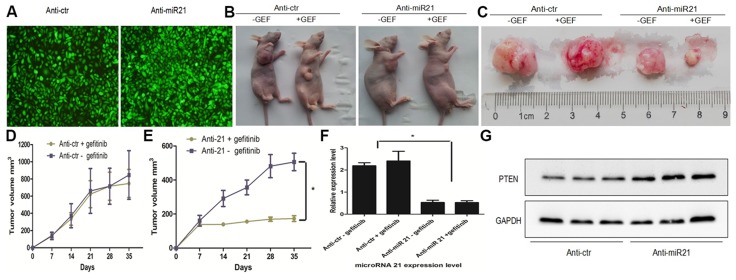
Anti-miR-21 restored gefitinib sensitivity of pc-9/GR *in vivo*. (A) Fluorescent pictures of pc-9/GR cells transfected by inhibitors of control miRNAs (ctr) or miR-21. (B) Representative tumor engraftments in nude mice injected with pc-9/GR cells stably transfected with anti-Ctr, anti-21. 35 days after injection and following treatment with vehicle (0.1% tween 80) or gefitinib (200 mg/kg), mice were sacrificed. The images show one representative mouse in each category. (C) Comparison of engrafted tumors in nude mice injected with pc-9/GR cells stably transfected with inhibitors of control miRNAs (ctr) or miR-21. The images show representative tumors in each category. (D) Tumor growth curve upon implantation of pc-9/GR+anti-Ctr cells with vehicle or gefitinib. (E) Tumor growth curve upon implantation of pc-9/GR+anti-miR-21 cells with vehicle or gefitinib. (F) qRT-PCR showing miR-21 down-regulation in pc-9/GR xenografts infected by anti-miR-21 compared with pc-9/GR cells infected by anti-Ctr. *P<0.01. (G) Pten protein levels in xenograft tumors analyzed by western-blot.

To analyze the role of miR-21 in modulating gefitinib sensitivity *in vivo*, we stably transfected pc-9/GR cells with GFP lentivirus constructs containing full-length anti–miR-21 or anti-Ctr **(** **Fig. 6A** **)**. PC-9 GR/anti-miR-21 or PC-9 GR/anti-Ctr were subcutaneously injected into the posterior flank of nude mice (n = 6). The tumor xenografts occurred 7 days after injection and nude mice with PC-9 GR/anti-miR-21 or PC-9 GR/anti-Ctr were treated with vehicle (0.1% tween 80) or gefitinib (200 mg/kg), respectively. Xenograft tumor volumes were measured every 7 days when palpable. Mice were sacrificed 35 days after implantation, and xenografts were collected and weighed. Representative nude mice and xenograft tumors are shown in Fig. 6B and Fig. 6C. We found that knockdown of miR-21 in pc-9/GR cells resulted in sharp inhibition of tumor growth and increased sensitivity to gefitinib in nude mice after 2 weeks of treatment (Fig. 6E) while in the anti-Ctr group, pc-9/GR cells still displayed gefitinib resistance properties (Fig. 6D). We confirmed the down-regulation of miR-21 in the xenograft tumors transfected by anti-miR21 by qRT-PCR (Fig. 6F). Total protein levels in representative tumor samples were analyzed by both Western blot and IHC test. We observed that miR-21 suppressed Pten expression in vivo by WB(Fig. 6G) and IHC (Fig. S2).

## Discussion

Gefitinib and erlotinib are the two EGFR-TKIs currently approved for the treatment of advanced NSCLC worldwide [29], [30], [31]. Gefitinib has been approved in Japan and worldwide based on the data from two large phase II trials of gefitinib monotherapy in previously treated patients with advanced NSCLC [IDEAL 1 and 2]. In IDEAL 1, Japanese patients gained particular clinical benefit from gefitinib treatment, with tumor response rate of 27.5% compared with 18.4% for the overall IDEAL 1 population [32], [33]. In the present study, among 46 patients with NSCLC receiving EGFR-TKIs, 12 patients had PR, 21 patients had SD, and 13 patients had PD; hence, the tumor response rate (CR+PR) was 26.09%, which was similar to the response rates of Japanese patients in IDEAL 1. The ISEL study showed that the patients treated with gefitinib had a higher tumor response rate compared with placebo (12.4 versus 2.1%, respectively), and the response rate was increased in patients of Asian origin [34]. The tumor response rates of gefitinib in NSCLC patients with EGFR mutation were similar: 83% in optimal study [35] compared with 62% in WJTOG3405 [36], 74% in NEJ002 [37], and 71% in IPASS [38]. In the present study, all 46 patients were of Asian origin and 44 patients had adenocarcinoma and hence, the response rates were higher compared with ISEL study. Three among 46 patients have survived more than 5 years and all of them had PR to EGFR-TKI treatment. Several biomarkers, including EGFR expression as assessed by immunohistochemistry (IHC), changes in EGFR copy-number detected by fluorescent in situ hybridization (FISH), and EGFR mutational status evaluated by sequencing or PCR, have been evaluated during some key trials [39]. Due to controversial results, they are not all validated as predictive biomarkers in NSCLC patients [10], [11]. However, a demonstrated relationship exists between EGFR mutational status and EGFR-TKIs sensitivity, making this the best predictor of clinical response to EGFR-TKIs [12], [13]. Activating mutations such as exon 21 missense point mutation L858R and in-frame deletions in exon 19 are the most commonly studied predictive biomarkers of response to EGFR-TKIs (gefitinib and erlotinib) [40], [41]. Development of resistance to TKI is a thorny problem in non-small cell lung cancer (NSCLC) treatment [42]. Despite the initial promising response to EGFR-TKIs in majority of NSCLC patients harboring sensitizing EGFR mutations [43], [44], most patients eventually relapse due to the emergence of acquired resistance such as the EGFR T790M mutation or MET amplification, both accounting for about 70% of the acquired resistance [4]. The challenge of tumor drug resistance therefore represents a barrier that confounds the ultimate goal of cure or long-term control of NSCLC.

Dysregulation of microRNAs (miRNAs) is a common feature in human cancers, including NSCLC [7]. Recent research has confirmed that miRNAs can modulate the multidrug resistance of many cancer cells; [45], [46], [47] for instance, Zhu et al [48] found that mir-181b modulates multidrug resistance by targeting Bcl2 in human cancer cell lines. Gao et al. found that mir-21 increases resistance to platinum-based chemotherapy in NSCLC [27]. Yang et al. reported that over-expression of miR-21 significantly decreased antiproliferative effects and apoptosis induced by cisplatin and induced cisplatin resistance by down-regulating Pten expression. [49] Bai et al. found that MicroRNA-21 regulates the sensitivity of diffuse large B-cell lymphoma cells to the CHOP chemotherapy regimen. [50] These findings demonstrate that miR-21 is active in regulating chemotherapy resistance. Rescent study revealed that miR-21 was involved in acquired resistance of EGFR-TKI in NSCLC which suggested a good reasoning for purusuing this investigation [51]. In this study, we first assessed miR-21 expression levels in 47 pairs of lung cancer tumor specimens and adjacent normal tissues and our results were in agreement with previous data showing up-regulation of miR-21 in lung cancer tumor tissues compared with adjacent normal tissues. [52], [53] As Pten is known to be one of the most important target gene of miR-21 in cancer development. [14], [54] In this study, Pten expression levels in lung cancer tissues were evaluated using WB and IHC analyses. The WB results revealed that the Pten protein levels were significantly reduced in tumor tissues (72.3%) compared with adjacent normal tissues. The rest of high Pten expression levels in tumors (27.7%) could possibly be due to some normal tissues within the tumor sample and the low expression levels of miR-21 in 21.3% of the tumors comparing with its adjacent normal tissues. The ratio was consistent with each other. The authors found an inverse correlation between miR-21 expression levels and IHC scores of Pten signals. These findings confirmed that up-regulation of miR-21 and down-regulation of Pten is a carcinogenic factor in NSCLC. Based on reports linking miRNA and regulatiion of drug resistance, [14], [47] we asked whether alteration of miR-21/Pten expression contributes in modulating TKI sensitivity of NSCLC. By analyzing miR-21/Pten expression levels and clinical response to TKI treatment in 46 NSCLC patients, we found that high miR-21 expression levels and low Pten protein levels were associated with poor clinical response to TKIs and shorter overall survival. Therefore, we hypothesized that miR-21/Pten expression alteration may constitute another biomarker of TKI sensitivity modulation. To test our hypothesis, we used pc-9 cells and the gefitinib resistant counterpart (pc-9/GR) in our *in vitro* studies. We observed miR-21 up-regulation and down-regulation of Pten in pc-9/GR cells compared with pc-9 cells. Although over-express miR-21 by mimic transfection demonstrates minimal resistance to gefitinib compared to the PC-9/GR resistant cell line (3B compared to 4B). We did find that either over-expression of miR-21 in pc-9 cells or knockdown of miR-21 in pc-9/GR cells could significantly reverse their TKI sensitivity.

Furthermore we demonstrated that miR-21 modulates gefitinib sensitivity in both pc-9 and pc-9/GR cells by down-regulation of Pten and activation of PI3K/AKT and ERK signaling pathways. The PI3K/AKT and ERK pathways play crucial roles in gefitinib sensitivity regulation [55], [56], [57]. Indeed, persistent activity of the PI3K/Akt and/or Ras/Erk pathways is associated with gefitinib-resistance of NSCLC cell lines. [58] Janmaat et al. described Gefitinib-resistant NSCLC cell lines showing EGFR-independent activity of PI3K/Akt or Ras/Erk pathways. [59] Preclinical studies also demonstrated that continued activation of downstream signaling pathways, especially PI3k/AKT, is sufficient to confer resistance to EGFR-TKI by bypassing the EGFR blocking, [60] consistently with our findings described above. To further validate the role of miR-21 in regulating TKI resistance, miR-21 knockdown was performed in the xenograft model. In agreement with our *in vitro* data, the *in vivo* results showed that knockdown of miR-21 resulted in sharp inhibition of tumor growth and reserved gefitinib sensitivity of pc-9 GR in nude mouse model.

In summary, these findings suggest that miR-21 functions as a carcinogenic factor by negatively regulating Pten expression in human NSCLC tissues. miR-21 levels inversely correlate with protein levels of Pten and high miR-21 expression levels are associated with shorter DFS. High miR-21/low Pten expression levels may indicate a poor TKI clinical response in patients taking TKI treatment. In *in vitro* assays, we found miR-21 up-regulation accompanied with down-regulation of Pten in pc-9/GR cells relatively to pc-9 cells. Moreover, over-expression of miR-21 significantly decreased gefitinib sensitivity by down-regulation of Pten expression and activation of Akt and ERK pathways, while knockdown of miR-21 dramatically restored gefitinib sensitivity of pc-9/GR cells by up-regulation of Pten expression and inactivation of AKT and ERK pathways both *in vivo* and *in vitro*. These data suggest that miR-21/Pten expression alteration constitutes a novel mechanism for understanding TKI resistance in NSCLC and provide a new basis for the use of miR 21/Pten-based therapeutic strategies for reversing gefitinib resistance in NSCLC.

## Supporting Information

Figure S1 **The mRNA levels and protein levels of PTEN determined by real-time PCR (A) and Western-blotting (B) in PC-9/GR cells 60 h after being transfected with siPTEN#1, siPTEN#2, or scramble siRNA(siSCR).** Results are presented as mean ± SD from three replicate experiments. *indicates the significant difference when compared to the control (P<0.05), **indicates the significant difference when compared to the control (P<0.01).
(JPG)

Figure S2 **Pten protein levels in xenograft tumors analyzed by IHC.**
(JPG)

Table S1 **Clinicopathological data for the 47 NSCLC samples.**
(DOC)

Table S2 **Clinical, pathologic, category of TKI and response to TKI therapy in the 46 studied patients.**
(DOC)
