# Supplementary figures and images for: Alteration in Mir-21/PTEN Expression Modulates Gefitinib Resistance in Non-Small Cell Lung Cancer
Source: PLoS One. 2014 Jul 24;9(7):e103305. doi: 10.1371/journal.pone.0103305 (PMC4110008; doi:10.1371/journal.pone.0103305)

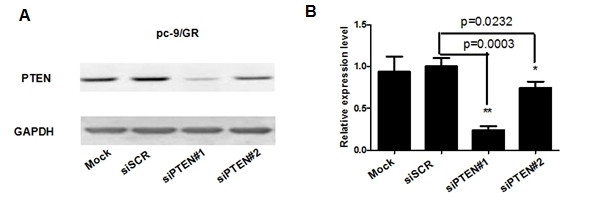

Supplement: Figure S1 — The mRNA levels and protein levels of PTEN determined by real-time PCR (A) and Western-blotting (B) in PC-9/GR cells 60 h after being transfected with siPTEN#1, siPTEN#2, or scramble siRNA(siSCR). Results are presented as mean ± SD from three replicate experiments. *indicates the significant difference when compared to the control (P<0.05), **indicates the significant difference when compared to the control (P<0.01). (JPG) [file pone.0103305.s001.jpg]

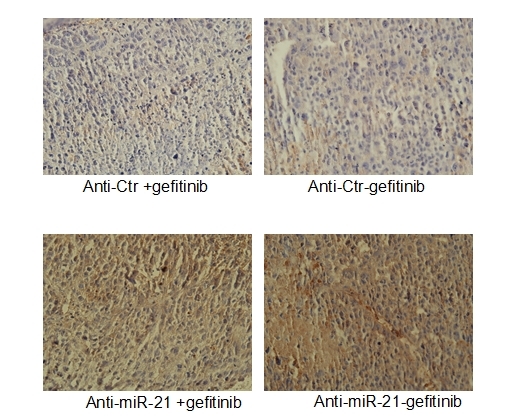

Supplement: Figure S2 — Pten protein levels in xenograft tumors analyzed by IHC. (JPG) [file pone.0103305.s002.jpg]
