# Supplementary material for: Alteration in Mir-21/PTEN Expression Modulates Gefitinib Resistance in Non-Small Cell Lung Cancer
Source: PLoS One. 2014 Jul 24;9(7):e103305. doi: 10.1371/journal.pone.0103305 (PMC4110008; doi:10.1371/journal.pone.0103305)
Supplement: Table S1 — Clinicopathological data for the 47 NSCLC samples. (DOC) [file pone.0103305.s003.doc]

**Supplement Table 1**. Clinicopathological data for the 47 NSCLC samples

| **Characteristic** | **Value** |
| --- | --- |
| **Sex** |  |
| Male | 38 |
| Female | 9 |
| **Age, years** |  |
| Mean±SD | 63.98±9.50 |
| Range | 44~80 |
| **Histological subtype** |  |
| Adenocarcinoma | 20 |
| Squamous cell carcinoma | 16 |
| adenosquamous carcinoma | 1 |
| **Grade** |  |
| I | 18 |
| II | 15 |
| III | 14 |
| **Stage** |  |
| IA | 10 |
| IB | 10 |
| IIA | 8 |
| IIB | 12 |
| IIIA | 5 |
| IIIB | 1 |
| IV | 1 |
