# Supplementary material for: Alteration in Mir-21/PTEN Expression Modulates Gefitinib Resistance in Non-Small Cell Lung Cancer
Source: PLoS One. 2014 Jul 24;9(7):e103305. doi: 10.1371/journal.pone.0103305 (PMC4110008; doi:10.1371/journal.pone.0103305)
Supplement: Table S2 — Clinical, pathologic, category of TKI and response to TKI therapy in the 46 studied patients. (DOC) [file pone.0103305.s004.doc]

**Supplement Table 2**. Clinical, pathologic, category of TKI and response to TKI therapy in the 46 studied patients.

| **Characteristic** | **Category of TKI** | |
| --- | --- | --- |
| **Sex, no** | Gefitinib (33) | Erlotinib (13) |
| Male | 12 | 7 |
| Female | 21 | 6 |
| **Age, years** |  |  |
| Mean±SD | 62.58±1.66 | 59.62±3.16 |
| Range | 46~82 | 44~77 |
| **Histological subtype** |  |  |
| Adenocarcinoma | 31 | 13 |
| Squamous cell carcinoma | 2 | 0 |
| **Grade** |  |  |
| I | 5 | 1 |
| II | 15 | 6 |
| III | 13 | 6 |
| **Stage** |  |  |
| IA | 7 | 4 |
| IB | 5 | 1 |
| IIA | 4 | 1 |
| IIB | 6 | 2 |
| IIIA | 6 | 4 |
| IIIB | 3 | 0 |
| IV | 2 | 1 |
| **Clinical response** |  |  |
| PR | 6 | 6 |
| SD | 17 | 4 |
| PD | 10 | 3 |
